# Design process of the nanofluid injection mechanism in nuclear power plants

**DOI:** 10.1186/1556-276X-6-363

**Published:** 2011-04-27

**Authors:** Myoung-suk Kang, Changhyun Jee, Sangjun Park, In Choel Bang, Gyunyoung Heo

**Affiliations:** 1 Kyung Hee University, 1 Seocheon-dong, Giheung-gu, Yongin-si, Gyeonggi-do 446-701, Republic of Korea; 2 Ulsan National Institute of Science and Technology, 100 Banyeon-ri, Eonyang-eup, Ulju-gun, Ulsan-si 689-798, Republic of Korea

## Abstract

Nanofluids, which are engineered suspensions of nanoparticles in a solvent such as water, have been found to show enhanced coolant properties such as higher critical heat flux and surface wettability at modest concentrations, which is a useful characteristic in nuclear power plants (NPPs). This study attempted to provide an example of engineering applications in NPPs using nanofluid technology. From these motivations, the conceptual designs of the emergency core cooling systems (ECCSs) assisted by nanofluid injection mechanism were proposed after following a design framework to develop complex engineering systems. We focused on the analysis of functional requirements for integrating the conventional ECCSs and nanofluid injection mechanism without loss of performance and reliability. Three candidates of nanofluid-engineered ECCS proposed in previous researches were investigated by applying axiomatic design (AD) in the manner of reverse engineering and it enabled to identify the compatibility of functional requirements and potential design vulnerabilities. The methods to enhance such vulnerabilities were referred from TRIZ and concretized for the ECCS of the Korean nuclear power plant. The results show a method to decouple the ECCS designs with the installation of a separate nanofluids injection tank adjacent to the safety injection tanks such that a low pH environment for nanofluids can be maintained at atmospheric pressure which is favorable for their injection in passive manner.

## Introduction

One of the methods for enhancing the safety of nuclear power plants (NPPs) is related to improve their heat transfer capability. Nanofluids are known to exhibit superior heat transferability and are therefore being actively investigated for engineering applications [1-4]. Recently, the studies on the introduction of nanofluids for emergency core cooling systems (ECCSs) which is one of engineered safety features of NPPs were conducted [5-7]. Such works were characterized by the enhancement of critical heat flux (CHF) via nanofluid injection in cases of loss of coolant accidents (LOCAs). While taking the benefits of nanofluid injection during accident conditions, it is apparent that the nanofluid-engineered ECCSs should be compatible with conventional systems during normal operations to make nanofluid technologies practical in NPPs. With this motivation, it is, therefore, important to analyze the functional requirements (FRs) for integrating the ECCS and nanofluid injection mechanism without loss of performance and reliability of the conventional systems.

This study employs axiomatic design (AD) and TRIZ for analysis of FRs and creation of a relevant nanofluid-engineered ECCS. The theory underlying AD is based on the hypothesis that superior design begins with certain axioms that facilitate the creation of systems through interactive mapping of FRs and design parameters (DPs). In addition, AD facilitates reasonable and logical steps that take conceptual design processes into account [8,9]. The second tool employed in this study TRIZ, which is a Romanized acronym of a Russian phrase meaning, 'theory of solving inventor's problems' or 'theory of inventive problem solving' [10]. The TRIZ theory applies abstraction and concretization processes to facilitate the creation of solutions for problems recognized from an existing design by the AD process [11].

In this study, the analysis of a conventional design of an ECCS and several design alternatives to adopt nanofluid injection mechanism using the principles of AD has been presented, and also the compatibility of FRs and potential design vulnerabilities are discussed. The methods to enhance such vulnerabilities are drawn from TRIZ and finally concretized a specific design of a nanofluid-engineered ECCS. In this article, a conceptual design incorporating nanofluids for the ECCSs of the Korean Advanced Power Reactor 1400MWe (APR1400) has been elicited.

## Background

### LOCA and ECCS

A LOCA is an accident which occurs due to a break in a reactor coolant system (RCS) pipeline in a NPP. LOCAs are considered to be serious accidents because of the possibility of core meltdown. As the reactor coolant drains out of the RCS, the temperatures of the nuclear fuel rods increase due to the lack of coolant. Core meltdown may result from the increased temperatures of the fuel rods.

An ECCS is one of the engineered safety features and supplies sufficient coolants to a core for maintaining fuel temperatures below its melting point and therefore core meltdown could be avoided in case of a LOCA. An ECCS consists of a safety injection system (SIS) and a shutdown cooling system (SCS). The purpose of the SIS is core heat removal and power decrease via borated water injection following a LOCA. In the APR1400, emergency coolant is injected from safety injection tanks (SITs) and via safety injection pumps (SIPs) from an in-containment refueling water storage tank (IRWST). The SCS is designed to provide residual heat removal in shutdown situations, which is a long term operation mode [12]

### Nanofluids

Nanofluids are engineered colloidal dispersions with a traditional coolant as a base in which nanoparticles are suspended. In 1995, Choi, who first named nanofluids, published the results of his theoretical research. Subsequent developments in nanofluid engineering have contributed to the rapid growth in nanotechnology and surface technologies over the last 10 years [2].

The colloidal suspensions have substantially shown intriguing thermal performances regarding four points: (1) increased thermal conductivity (approx. 150%), (2) increased single-phase heat transfer coefficient (approx. 60%), (3) increased critical heat flux with extended nucleate boiling regime (approx. 200%), and (4) improved quenching efficiency. Although there is a lack of agreement of the experimental data in the literature and a lack of understanding of the physical mechanisms describing nanofluid thermal performances, the present work was motivated by the fact that a nanofluid formulation could not be tailored to show the desired properties for nuclear systems if we do not consider it together with nuclear safety systems characterized inherently by various couplings in a system engineering [13]. These properties were expected to be better especially when nanofluids are employed as coolants in ECCSs, and several applications to light water reactors have been published [4-7]. The design alternatives suggested in previous studies can be summarized as follows:

OPTION 1: injection of nanofluids from conventional SITs

OPTION 2: installation of a nanofluids-engineered SIT which is dedicated for nanoparticle injection

OPTION 3: injection of nanofluids via IRWST lines

### Axiomatic design and TRIZ

The purpose of AD is to create the improved design of various tangible and intangible products. It provides an objective means for evaluating competing designs and enabling a better design to be chosen. The following two axioms are expressions that integrate common principles mapping FRs and DPs [9]:

Axiom 1: Independence Axiom. Maintain the independence of the FRs.

Axiom 2: Information Axiom. Minimize the information content of the design.

The mapping process between two domains can also be represented mathematically in terms of characteristic vectors that define the design goals or FRs and design solutions or DPs:

where [A] is the design matrix.

In order to satisfy axiom 1, [A] must be either diagonal or triangular. In the case where [A] is diagonal, each of the FRs can be satisfied independently by one of the DPs. This situation is called an uncoupled design. In the case where [A] is triangular, the independence of the FRs can be satisfied by determining the DPs in the proper sequence. This is called a decoupled design. In this case, [A] does not necessarily have to be triangular in strict sense. A design corresponding to any other form of the design matrix is said to be coupled. Consequently, a diagonal or triangular design matrix gives rise to a better design. In practice, however, most designs are coupled, so that designers must decouple the object design by intelligently selecting relevant DPs, which can be referred as an improved design. Finally, axiom 2 can be used for determining the best solution among the decoupled or uncoupled design options.

Following the AD process, designers may encounter a problem about how to obtain a decoupled solution or convert a coupled design into decoupled. TRIZ is one of the tools to give a reasonable approach to find such a solution. TRIZ is a knowledge-based methodology for facilitating problem-solving in the context of invention. Specifically, by utilizing problem-solving elements from prominent inventions, one can solve one's own problems or produce further innovations with less effort. TRIZ is based on a hypothesis that patterns or analogical concepts leading to exceptionally innovative solutions to technical problems can be extracted through analysis of past inventions. In support of the hypothesis, an enormous number of patents have been researched and still being analyzed [10].

**Figure 1:**
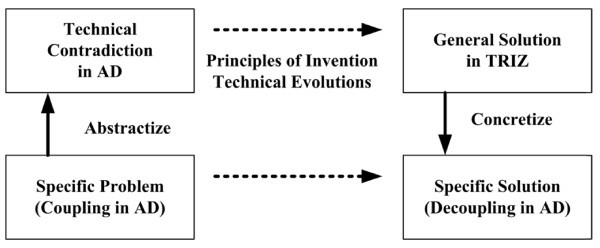
**Problem-solving using TRIZ in AD process**.

The most significant benefit of using TRIZ in AD process is identification and consequently removal of technical contradiction (which is the interchangeable term as coupling in AD process) with the help of certain principles. It should be noted that decoupling a design in terms of AD is similar to removing a technical contradiction in TRIZ. In a design process, the principles of AD do not practically provide a guaranteed method to determine the proper DPs. However, all of the TRIZ principles focus on methods to exclude technical contradictions within not only all branches of engineering, but also non-technical fields as well. Figure 1 shows how to apply TRIZ in AD to a problem.

The coupling recognized in AD is equivalently associated with a contradiction with TRIZ. To create the DPs to decouple the design matrix, designers should represent their specific problems with typical language. This process, called 'abstractization', is the first step for applying the TRIZ on the results of AD process. Utilizing the TRIZ principles for abstractized problems or contradictions helps designers obtain general solutions which are the ideas to remove the technical contradictions by changing the physical parameters such as geometrical shapes, physical conditions, matter states, and so on. Since the general solutions are not directly applicable to the specific problems, mostly designers are required to concretize the general solutions for the specific solutions concerning the characteristics of the original problems [14,15].

## Design process and results

The design process was started from the reverse engineering of conventional ECCS and nanofluids injection mechanisms using the principles of AD followed by identification of design weaknesses in terms of couplings. These design weaknesses was then eliminated by virtue of TRIZ, and finally a specific design was created.

### Reverse engineering of conventional ECCSs

We investigated the FRs of the ECCS of the APR1400 to employ a nanofluid injection mechanism. Three nanofluid injection mechanisms were referred from previous studies [5-7]. The FR and DP decomposition starts at the top requirement of the ECCS as follows [14,15]:

FR0: shut down a reactor while preventing core melt after LOCA

DP0: nanofluid-engineered ECCS

The sub-FRs of DP0 are the requirements to inject coolant during the initial phase (FR1) and to provide long-term cooling (FR2). The respective DPs of FR1 and FR2 constitute the SIS and SCS as follows:

FR1: injection of coolant at the beginning of LOCA

DP1: safety injection system

FR2: provides long-term cooling for reactor cold shutdown

DP2: shutdown cooling system

FR1 should be related to the reflooding and refilling stage. The corresponding DP1 is primarily associated with all options. With regard to option 1, FR1.1, FR1.2, and FR1.3 are as follows:

FR1.1: provides coolant from conventional SITs

DP1.1: injection of borated water from conventional SITs

FR1.2: provides nanoparticles

DP1.2: [Option 1] injection of nanofluids from conventional SITs as nanoparticles mixed with borated water

FR1.3: provides coolant from a conventional IRWST

DP1.3: injection of borated water from conventional IRWST

We can define the sub-requirements of DP1.1 again as follows.

FR1.1.1: provides sufficient coolant inventory

DP1.1.1: conventional SITs

FR1.1.2: provides a driving force for coolant

DP1.1.2: pressurized nitrogen

FR1.1.3: provides a control signal for coolant

DP1.1.3: passive actuation

FR1.1.4: provides a flow path for coolant

DP1.1.4: valve arrangement

A sub-FR of DP1.2, 'injection of nanofluids from conventional SITs as nanoparticles mixed with borated water' is required to provide a sufficient quantity of nanoparticles, stability of the nanoparticles, convenient nanoparticle sampling, compatibility with tanks, structures for nanoparticle storage, and continuous mixing with borated water. They may be summarized is as follows:

FR1.2.1: provides a sufficient quantity of nanoparticles

DP1.2.1: volume percentages of nanoparticles

FR1.2.2: provides continuous stability of nanoparticles

DP1.2.2: pH stabilizer (acidic condition)

FR1.2.3: provides a check for nanoparticle stability

DP1.2.3: sampling device

FR1.2.4: provides compatibility for corrosion resistance

DP1.2.4: neutral pH stabilizer

FR1.2.5: provides storage for nanoparticles

DP1.2.5: conventional SIT

FR1.2.6: mixes the nanoparticles and coolant

DP1.2.6: preloaded nanoparticles into a conventional SIT

**Figure 2:**
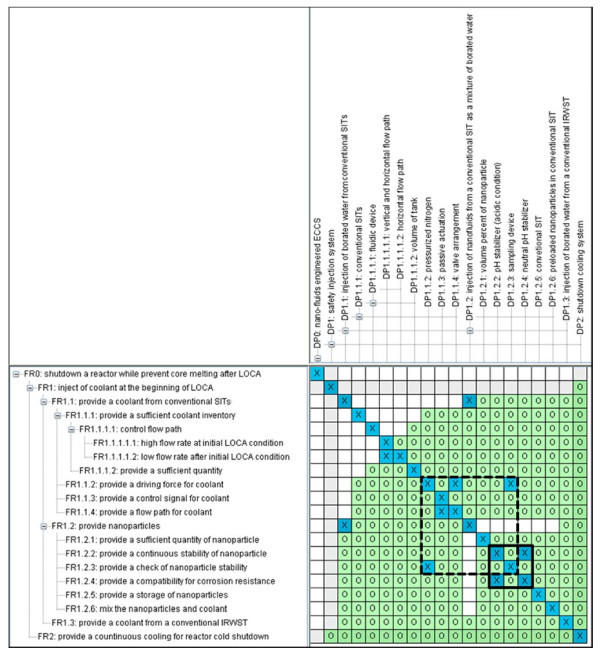
**Design matrix for option 1 (dash: coupling 1, solid: coupling 2)**.

We completed the set of FRs and DPs and Figure 2 shows the entire design matrix for option 1. The boxes marked in Figure 2 indicate that the functional couplings or technical contradictions. There are two important couplings identified due to the share of the conventional SIT as a reservoir of coolant and nanofluid:

where FR1.1.2 provides a driving force for the nanoparticles, FR1.2.3 provides a check for nanoparticle stability, andDP1.1.2 pressurized nitrogen, DP1.2.3 sampling device.

This is the first contradiction caused by nitrogen pre-pressurization and nanofluid sampling. The sampling for checking the stability of nanoparticles requires depressurization of the contents of the SIT. However, the depressurization of the SIT does not contribute to a driving force for the coolant and nanoparticles.

where FR1.2.2 provides stability of nanoparticles, FR1.2.4 provides compatibility for corrosion resistance, DP1.2.2 pH stabilizer (acidic condition), and DP1.2.4 neutral pH stabilizer.

The original option 1 does not mention any methods to control corrosion caused by nanofluids. To ensure homogeneity of the nanoparticles in the SIT, the pH state of the SIT should be acidic. However, acidic contents can induce corrosion of the SIT. Consequently, the FRs and DPs which satisfy each of these functions contradict one another.

Using the same method, option 2 was then analyzed. Identifying the FRs and DPs for option 2 is similar to the process for option 1 except the facts that option 2 uses a separated nanofluid injection tank and the tank has titanium coating due to the corrosion of the tank wall. Since the size of the nanofluid injection tank is much smaller than the SITs, the wall coating is reasonable in terms of cost.

FR1.1: provides borated water

DP1.1: conventional SITs

FR1.2: provides nanoparticles

DP1.2: [Option 2] a nanofluid engineered SIT which is dedicated for nanoparticle injection

FR1.3: provides coolant from a conventional IRWST

DP1.3: injection of borated water from a conventional IRWST

**Figure 3:**
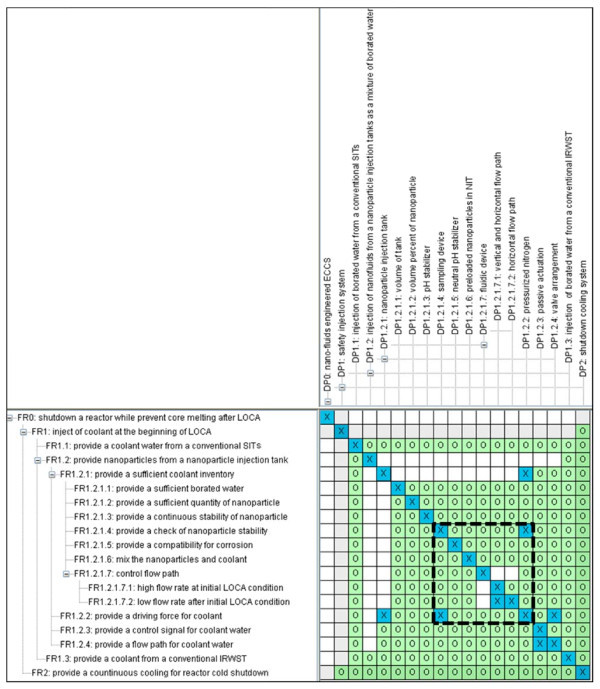
**Design matrix for option 2**.

Figure 3 shows the coupling in designing option 2. The coupling in option 2 is identical to the first coupling of option 1 since it also needs nitrogen pre-pressurization and nanofluid sampling.

**Figure 4:**
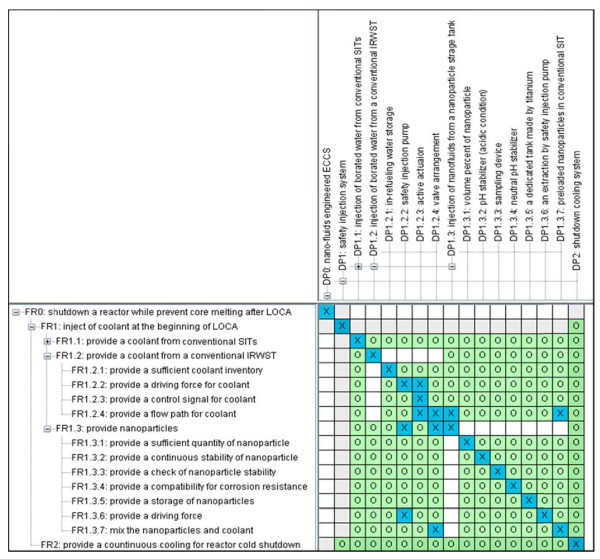
**Design matrix for option 3**.

Option 3 is the nanoparticle injection method connected with the IRWST. It provides the nanoparticles in a separate tank with coolant from the IRWST in a mixed state by the SIP. Figure 4 shows the resulting design matrix for option 3.

In option 3, any significant couplings do not exist due to a separate reservoir for nanofluid and an active driving force such as SIPs.

### Considerations for constraints

In order to finalize a design process, we should consider design constraints, for example, injection time, reliability, installation space, and cost for nanofluid-engineered ECCSs. The constraints belong to a detailed design process so it is not easy to estimate them quantitatively at this moment. We discuss the overall aspects of constraints on the basis of the analyzed FRs.

First, nanoparticles should be promptly injected into the core. From this viewpoint, options 1 and 2 are preferable because the SITs can be activated at an early stage of LOCAs. Option 3 may contribute to coolability at later stage. On the other hand, options 1 and 2 do not further provide nanofluids after the refill stage. Option 3 can provide a continuous nanofluid via a flow rate control device connected to the IRWST. Second, nanoparticles must be provided reliably in any types of injection mechanism. Option 1 can be regarded as the best from the viewpoint of risk due to its passive characteristic. Option 2 and 3 may need additional devices to isolate and pass the nanofluids in the tank in an appropriate manner, so reliability may be less than option 1. Third, option 1 does not require any additional space in the containment for installation as discussed. In practice, a large space may not be required for installation of a nanoparticle injection tank for option 2 and 3 because the volume of nanofluids is much smaller than that of emergency coolant. From the viewpoint of cost, installation space, additional manufacturing, maintenance may be associated. Since option 2 and 3 equips separate devices, it is likely to need higher cost.

### Design modification

In this section, we reformed conventional nanoparticle injection mechanisms using TRIZ. First, we focused on the coupling discussed for option 1 and 2. For the first coupling, consisting of the FRs 'provide a driving force for the nanoparticles' and 'provide a check of nanoparticles stability' and the DP 'pressurized nitrogen' and 'sampling device', we abstractized the technical contradiction as 'pre-pressurization, but depressurization'. Then, we applied the principles of TRIZ to find the general solution of the technical contradiction with a contradiction matrix system. This matrix provides selective options for 'Features to improve' and 'Undesired results'. We applied our technical contradiction to this matrix by the feature to improve, 'the difficulty of detection and measurement' and the undesired result, 'the Shape'. The result of this matrix is summarized in Table 1.

**Table 1:** Inventive principles in terms of the first coupling

Inventive principles	Description of inventive principles
1. Cheap short-living	Replace an expensive object with a multitude of inexpensive objects, compromising certain qualities
2. The other way around	Invert the action used to solve a problem. For example, instead of cooling an object, heat it. Make the movable parts fixed, and the fixed parts movable Turn the object 'upside down'
3. Segmentation	Subdivide an object into parts Make an object easy to disassemble Increase the degree of fragmentation of an object

4. Inert atmosphere	Replace a normal environment with an inert one Add neutral parts, or inert additives to an object

We took the third and fourth principles in this case, though all principles in the results can be allowable. Considering the third principles, the conventional SITs and nanofluid injection tank should be separated. Then, by the fourth principle, SITs should be pressurized and the nanofluid injection tank should keep depressurized but can pressurize depending on conditions. At this time, the second coupling in option 1 was simultaneously resolved by adopting a titanium coating tank for nanofluids, which is the same method for option 2.

**Figure 5:**
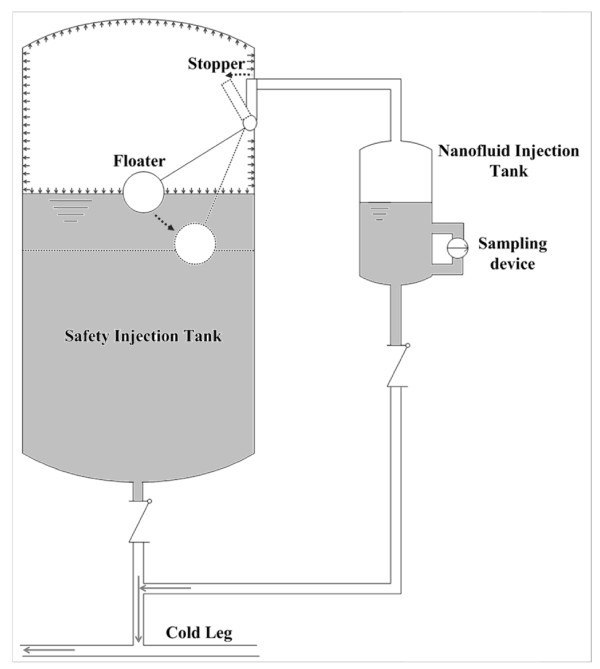
**Schematic diagram of nanofluid-engineered SIT (out of scale)**.

Finally we concretized a nanofluid-engineered ECCS with a separate nanofluid injection tank as shown in Figure 5.

The developed nanofluid-engineered ECCS consists of a borated water storage and a nanofluid storage. The borated water storage tank, which is the conventional SIT, can sustain a pressure of over 40 bars. On the other hand, the nanofluid storage tank can maintain its contents at atmospheric pressure, while also maintaining them at a stable low pH with a titanium coating on the inner surface of the tank, which is required to tackle the nanoparticle stability problem.

As shown in Figure 5, during normal operation, the borated water and nanofluids are separated by an isolation stopper connected to a floater inside the SIT. This isolation stopper is placed at the inner surface of the SIT by pressurized nitrogen and the floater with sealing the pipe line connecting the SIT and nanofluids injection tank. Following a LOCA, the pressure inside the RCS decreases below the pressure of the SITs, at which point emergency coolant is injected into the RCS. At this moment, the gravitational force generated by the floater opens the isolation stopper. Finally, the pressurized nitrogen forces the nanofluids, mixed with coolant from the SITs, to be injected. The decoupled matrix resulting from this design is as follows:

where FR1.1.2 provides a driving force for the nanoparticles, FR1.2.3 provides a check for nanoparticle stability, DP1.1.2 pressurized nitrogen, and DP1.2.3 sampling device.

where FR1.2.2 provides stability to the nanoparticles, FR1.2.4 provides compatibility for corrosion resistance, DP1.2.2 pH stabilizer (acidic condition), and DP1.2.4 coating of anti-corrosion material in nanoparticle storage tank.

From the design matrix, the sampling device of the new system is no longer disturbed by pressurized nitrogen. Likewise, acidic condition of coolant will not harm to the tank anymore.

## Conclusions

The history of nuclear industries should be the same as the history of nuclear safety. All activities from design to maintenance of NPPs are associated with safety. Even though the benefit of nanofluids is apparent in terms of heat transfer capability, its application should be considerate. It should be noted that both experience and theory have shown that the conceptual design stage is the most important for system's performance as well as safety, particularly in complex systems. Therefore, this study was performed to motivate and accelerate the use of nanofluid technologies in the practical applications.

The new method of nanofluids injection under LOCAs was proposed in this paper with analyzing the FRs of the candidates suggested in the previous researches on the basis of the principles of AD and TRIZ tools. Following the analysis of conventional ideas, major couplings were recognized. To solve these kinds of couplings, the installation of a separate nanofluids injection tank adjacent to the SITs was proposed. This tank is connected to the SIT with a passive gravity-operated stopper. The interior of the nanofluids injection tank is titanium-coated to permit a low pH environment. Depending on the detailed design processes, some of DPs may be revised or replaced, but this paper will contributed on the development of the preliminary steps connecting a scientific phase and an engineering phase.

## Abbreviations

AD: axiomatic design; CHF: critical heat flux; DPs: design parameters; ECCSs: emergency core cooling systems; FRs: functional requirements; IRWST: in-containment refueling water storage tank; LOCAs: loss of coolant accidents; NPPs: nuclear power plants; RCS: reactor coolant system; SCS: shutdown cooling system; SIS: safety injection system; SIPs: safety injection pumps; SITs: safety injection tanks.

## Competing interests

The authors declare that they have no competing interests.

## Authors' contributions

MK participated in the reverse engineering and drafted the manuscript. CJ performed the design modification and participated in the reverse engineering. SP performed the precedent analysis and participated in the reverse engineering. IB provided the properties of nanofluids. GH conceived of the study, and participated in its design and coordination. All authors read and approved the final manuscript.
